# Integrating system biology and intratumor gene therapy by trans-complementing the appropriate co-stimulatory molecule as payload in oncolytic herpes virus

**DOI:** 10.1038/s41417-024-00790-8

**Published:** 2024-06-05

**Authors:** A. Finizio, P. Pagano, A. Napolano, G. Froechlich, L. Infante, A. De Chiara, S. Amiranda, E. Vitiello, S. Totaro, C. Capasso, M. Raia, A. M. D’Alise, P. de Candia, N. Zambrano, E. Sasso

**Affiliations:** 1 Dipartimento di Medicina Molecolare e Biotecnologie Mediche, Università degli Studi di Napoli Federico II, Via Pansini 5, 80131 Napoli, NA Italy; 2 CEINGE Biotecnologie Avanzate Franco Salvatore S.C.aR.L., Naples, Italy; 3 Nouscom S.R.L., Rome, Italy; 4 ImGen-T Srl, Viale del Parco Carelli, Napoli, NA Italy

**Keywords:** Tumour immunology, Genetic vectors

## Abstract

Systems biology has been applied at the multi-scale level within the cancer field, improving cancer prevention, diagnosis and enabling precision medicine approaches. While systems biology can expand the knowledge and skills for oncological treatment, it also represents a challenging expedition due to cancer complexity, heterogeneity and diversity not only between different cancer indications, but also in its evolution process through space and time. Here, by characterizing the transcriptional perturbations of the tumor microenvironment induced by oncolytic, we aimed to rationally design a novel armed oncolytic herpes virus. We found that intratumor oncovirotherapy with HSV-1 induces T-cell activation signatures and transcriptionally activates several costimulatory molecules. We identified differentially expressed costimulatory receptors and binding partners, where inducible co-stimulators (ICOS) resulted in the potentially most beneficial targeted therapy. Through an ex-vivo transcriptomic analysis, we explored the potential of arming an oncolytic virus as a combination therapy strategy; in particular, we engineered a targeted herpes virus encoding ICOSL (THV_ICOSL), which resulted in a significant improvement in tumor size control compared to unarmed parental virus. Also, combination with a PD-1 inhibitor enhanced antitumor efficacy as predictable by upregulation of PD-1 and ligands pair (PD-L1/PD-L2) upon oncolytic virus injection. Generation of the human version of this virus encoding hICOSL orthologue effectively and specifically activated human T cells by triggering the ICOS pathway. Our data support the data-driven generation of armed oncolytic viruses as combination immunotherapeutic with checkpoint inhibitors.

## Introduction

Recent innovation in omics technologies has allowed the acquisition of large-scale bioinformatic cancer datasets at a relatively affordable price with unprecedent detail from entire genomes up to single cell level. Beyond diagnostics, -omics technologies can further enhance and speed up drug discovery [1, 2]. Regarding therapy and clinical translation, -omics datasets contain unique opportunities to understand and exploit molecular mechanisms for personalized and preventive medicine [3–5]. Currently, systems biology applied to cancer immunotherapy is mainly focused on identification of biomarkers for precision medicine, discovery of novel targets or unveiling unpredicted molecular mechanism of action of known molecules [6–9]. Additional interesting fields of systems biology applied to cancer immunotherapy also are the identification of drug resistance mechanisms to established novel therapies, and finding correlation of such tumor markers with given amenable targets for immunotherapy (e.g., HER2 with adenosine purinergic pathway) [10, 11]. All these examples support the concept of precision medicine that requires the delivery to tumors, such immunotherapeutic agent. Many recombinant cytokines and check point modulatory antibodies have been successfully tested in both preclinical models and in the clinics, although they also raised *several biosafety concerns*. As a relevant example, the cause of IL2 and IL12 setback is the toxic high concentration necessary to exert their effect at the site of action. This issue can be overcome by intratumor immune gene therapy, that can be achieved by encoding the immunomodulatory gene of interest as a nucleic acid therapeutic (e.g., RNA-LNP, DNA) and directly injected into the tumor mass [12]. This approach suffers from the limited persistence of the therapeutic translated protein due to dilution and “consumption” of the corresponding nucleic acid. To overcome both systemic toxicity problems, and limited availability of therapeutic genes of interest (GOI), oncovirotherapy can be exploited. By targeting and replicating solely in tumor cells, oncolytic viruses (OV) offer the unique opportunity to transform -omics data in intratumor therapeutic implementation to reshape immunosuppressive tumor microenvironment. Indeed, it is amenable to encode the GOI into the oncolytic virus genome under the control of a strong and/or tumor-restricted promoter to obtain a persistent and tumor-confined concentration of GOI products, while decreasing systemic toxic concentration [13, 14]. Since FDA approval of T-VEC in 2015, the field of OVs rapidly evolved with four OVs and one non-replicative adenovirus approved globally. Although talimogene laherparepvec (T-VEC) and Nadofaragene firadenovec (non-replicative Adenovirus) remain the only approved moieties by FDA, Teserpaturev has been approved in Japan for R/R glioblastoma therapy [15]. Systems biology could empower the field of OVs to both improve safety and enhance efficacy. Transcriptomic data can be exploited to identify cancer specific promoters or tumor downregulated miRNAs to respectively put viral genes under the control of tumor-active promoters and restrict viral genes translation in healthy tissues by engineering 3’UTRs with miRNA seed sequences [16–19]. The scientific community is focused on the generation of more powerful OVs and in novel combination therapies [20]. OV versatility has paved the way to next-generation arming strategies exploiting OVs as intra-tumoral gene-therapy carriers to express genes of interest [21, 22]. Traditionally, the most common GOI has been the cytokine GM-CSF, present as payload in most of OVs in clinical trials and into the clinically approved T-VEC (previously OncoVEX^GM-CSF^) [23, 24]. While the broad immune effect of GM-CSF has been robustly demonstrated, to date there are several immunomodulatory factors which could exert higher influence on the anti-tumor response [25]. The GOI delivery via the OVs system at the tumor site may avoid systemic toxicity while obtaining efficient intratumor concentration [25]. Beyond the obvious payloads (e.g., IL12, IL2, prodrug activators), such data-driven arming strategies also arise. Rivadeneira D.B and colleagues highlighted a metabolic insufficiency in tumor infiltrating lymphocytes (TILs) by scRNAseq after oncolytic vaccina virus treatment. These data paved the way to reinforce antitumor immune response by a next-gen VV ectopically expressing leptin adipokine [26].

The concept of “signal two” (TCR complex plus co-stimulatory receptor) plays a central role in the efficient activation of effector antitumor T-cells. For this reason, most of these costimulatory receptors have been targeted by agonist antibodies (e.g., 41BB, OX40, ICOS, CD40) thanks to their ability of unleashing immune responses through therapeutic combinations with classical inhibitory checkpoints [2]. Unfortunately, acute onset of immune-related adverse events (irAEs) (liver toxicity and cytokine storm symptoms) has often emerged as a limiting factor. Most of these costimulatory ligands have been encoded into many preclinical and clinical stage OVs to cut to the chase [22, 27]. In many datasets from preclinical or clinical tumor biopsies a decreased expression of these co-stimulatory receptors (e.g., CD137, OX40, CD27, and CD28) on T cells has been observed, questioning the usefulness of unbiased use of costimulatory antibodies [28, 29]. Expressing costimulatory ligands into OVs is frequent in onco-virotherapy despite, as mentioned before, the unbiased choice of costimulatory ligands might not be beneficial. This emphasizes the potential benefits of data-driven targeting of the right pathway in the context of perturbation induced by the candidate drug. Zamarin D. and Allison J. showed a significant upregulation of ICOS and CTLA4 in B16-F10 TILs following new castle disease virus (NDV) OV treatment allowing to rationally design an effective next-gen combination on NDV encoding ICOSL in combination with CTLA4 blockade [30]. Trans-complementing the appropriate costimulatory factors by encoding the matched payload into OVs is a promising approach in onco-virotherapy field. Recently, the single-cell RNA sequencing is an excellent perspective of OV-treated cancers sustaining the concept of encoding “the right one at the right time” to further improve the therapy [31, 32]. Oncolytic viruses (OVs) replicate and selectively kill cancer cells, leading to the release of tumor antigens in an immunologically favorable milieu enriched of Damage and Pathogens Associated Molecular Patterns (DAMPs and PAMPs) that activate Pattern Recognition Receptors (PRR) [33] leading to effective antigen presentation, T cell infiltration and activation [11]. While tumor infiltration after OV treatment has been well described, a suboptimal activation can arise from lacking complete and sustained stimulation. This, together with tumor immune suppressive stimuli, induces effector T cells to rapidly become anergic. To sustain T cell activation, we here aimed to identify the potentially most beneficial costimulatory immune checkpoint ligand to encode into an oncolytic herpes virus through a cheap NanoString tumor bulk transcriptomic profiling approach. We also predicted the potentially most beneficial combination with systemic check point blockade to increase anti-tumor efficacy, offering a rational for clinical translation.

## Results

### Data-driven generation of a costimulatory molecule armed Oncolytic Targeted Herpes Viruses

**Fig. 1:**
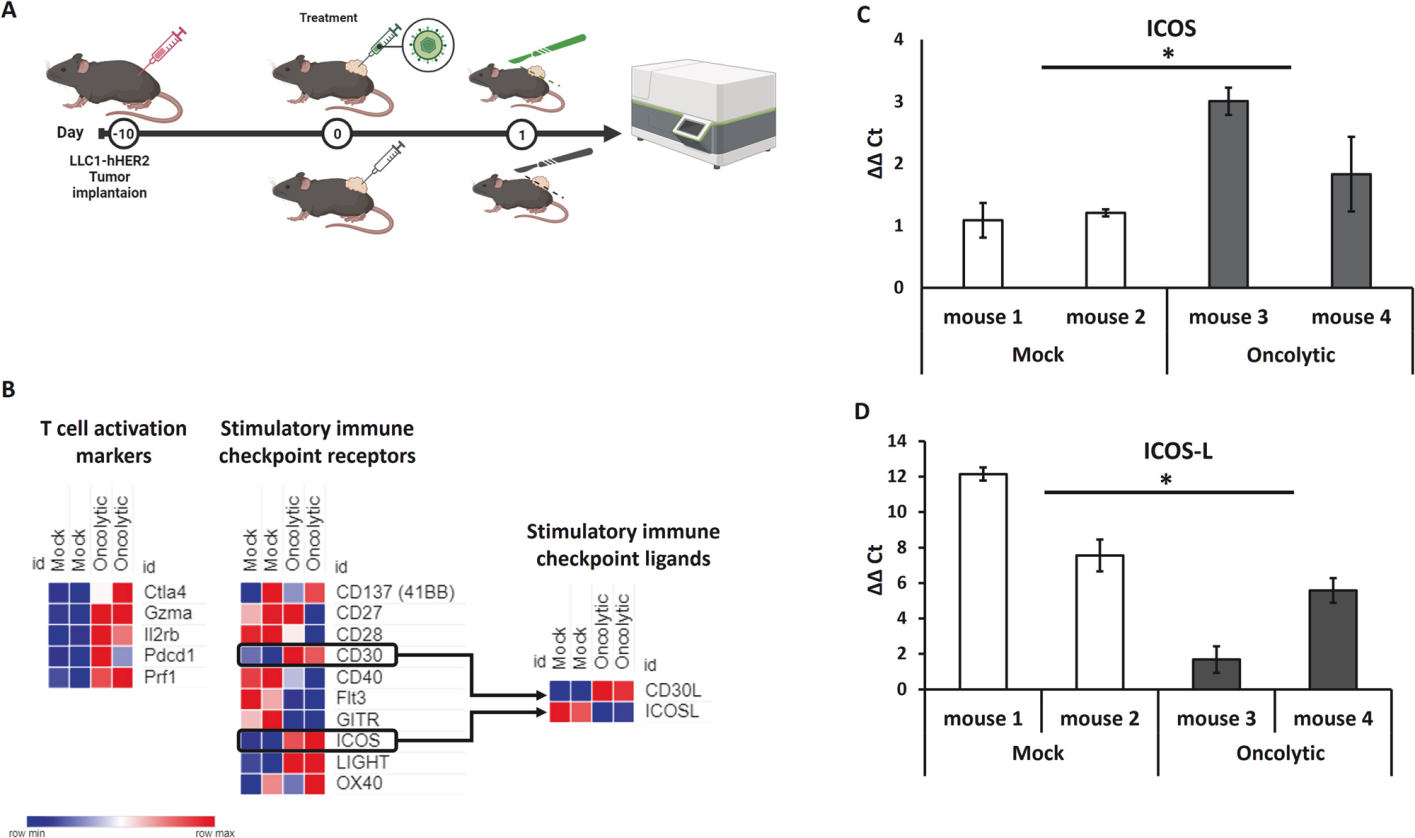
Analysis of tumor microenvironment perturbation after OV treatment. **A** Schematic representation of in vivo schedule of treatment. Subcutaneously implantation of LLC1-hHER2 into human HER2 tolerant mice. Mice were injected with R-LM113 OV or PBS. The day after injection, mice were killed, and tumor bulk RNA was analyzed by Nanostring Ncounter. **B** Funnel analysis of tumors immunological signature. Canonical activation markers were assessed and reported in the first set of heatmap. Only statistically significant differentially regulated genes were represented. Co-stimulus receptors were reported in the second set of heatmap. All the costimulatory receptors included in PanCancer immune profiling Nanostring panel were represented. Those resulting significantly differentially regulated were flagged by asterisk. Those statistically significantly upregulated (ICOS and CD30) were highlighted by black boxes. Matching ligand expression for CD30 and ICOS were analyzed and reported in the third panel of heatmaps. Broad institute’s Morpheus interface was used for heatmap representation. **C**, **D** Differential expression assessed by Nanostring was confirmed by Real-Time PCR from retrotranscribed cDNAs. One asterisk is used for *p* value < 0.005 for the heat map. In panels **C** and **D**, the asterisks represent *p* value < 0.05.

Co-stimulus of cytotoxic T cells is a fundamental step in immunity cycle that naturally happens when T cell encounters professional APCs. Interestingly, costimulatory ligands work also if ectopically expressed by non-professional cells, including cancer cells making them suitable for arming OVs. While tens of costimulatory ligand-receptor pairs have been described in different steps of immune activation cycle, the identification of the potentially most beneficial one as payload into a herpes-based oncolytic virus has never been attempted. To rationally choose the most appropriate co-stimulatory ligand, we aimed to assess transcriptomic perturbation upon OV treatment. We exploited an in vivo pilot study using an oncolytic herpes virus retargeted to tumor-associated antigen (TAA) HER2 (namely R-LM113). Briefly, this recombinant virus is generated by replacing a small moiety of viral glycoprotein D with a trastuzumab-derived single-chain antibody fragment targeting human HER2 [34]. This engineering contemporaneously allows de-targeting from endogenous HSV-1 entry receptors (i.e., HVEM, Nectin1) and re-targeting to HER2 as TAA model antigen to get tumor-restricted tropism. Murine LLC1 cells expressing human HER2 cells (LLC1-hHER2) were subcutaneously implanted into syngeneic immunocompetent human-HER2 tolerant C57BL6 mice. The use of hHER2 tolerant mice is necessary to avoid hHER2 recognition [35]. When tumor became established (>100 mm^3^), mice were randomized according to tumor size and received an intratumoral injection of the oncolytic virus. One day post injection was chosen as timepoint of analysis based on our previous report and on recent single cell seq clinical dataset that reveal how most of the transcriptional perturbations occur on day one post injection to then become less noticeable within one week [11, 31]. One day after treatment, mice were euthanatized, and tumors were excised. Bulk RNA was analyzed by PanCancer immune profiling panel by Nanostring NCounter and a funnel analysis was conducted to identify immune patterns to eventually reinforce by encoding payloads into the OV. Mock (PBS) injected mice were used as control (Fig.1A). We first ensured to identify a T cell activation signature as showed by significant upregulation of effector T cells markers (Fig.1B). We then analyzed the expression of costimulatory receptors to find out those with a significantly enhanced expression after OV treatment. Among TNF superfamily costimulatory receptors, both ICOS and CD30 resulted significantly upregulated after OV treatment. Restricting the analysis to these two receptors, we finally looked at the expression of matching ligands. While CD30L was strongly upregulated, ICOSL instead resulted downregulated after treatment (Fig.1B). As CD30 was already potentially activated by endogenous tumor microenvironment reshape, we decided to focus our efforts on ICOSL, and its expression, together with that of ICOS were validated by realtime PCR confirming Nanostring data (Fig. 1C, D).

**Fig. 2:**
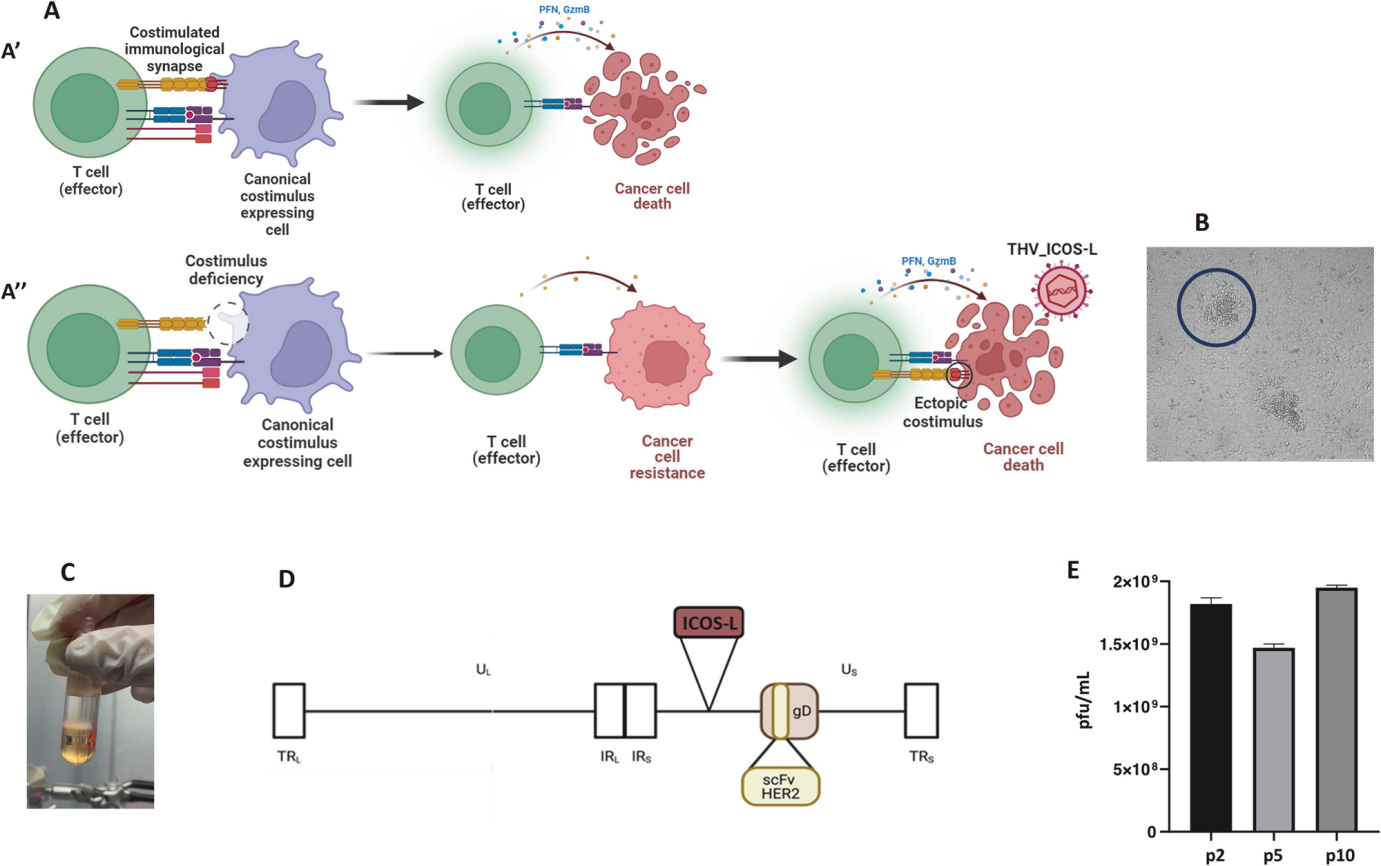
Generation of THV_mICOSL. **A** Schematic representation of immunological synapses required for fully competence of antitumor T cell response. **A′** T cell encounters a canonical costimulus-expressing cell leading to complete activation of T effector cell and cancer cell death. **A″** T cell effector encounters a cell which is deficient for the expression of costimulus; T cell is not completely activated. The ectopic expression of costimulatory ligands on the surface of tumor cells, mediated by oncolytic vectors, leads to a complete activation of T lymphocytes. (**B**) THV_ICOSL BAC DNAs were transfected in SKOV3 cells to produce infectious viral particles. Representative single plaque peaking. **C** Purification of THV_mICOSL through iodixanol gradient; the thick band indicates good productivity. **D** The murine ICOSL coding sequence was inserted into intergenic region US1-US2 in the background of R-LM113. The scFv targeting hHER2 is shown in glycoprotein D. © THV_mICOSL viral yield over ten passages (p2, p5 and p10). Differences in viral yield were not statistically significant. **E** Viral yield at different passages.

With the aim of compensating costimulus ligand deficiency, we modified R-LM113 virus to encode murine ICOSL to ectopically trans-complement ICOS function (Fig. 2A). An expression cassette including murine codon optimized mICOSL coding sequence under the control of CMV promoter and upstream a bovine growth hormone polyadenylation signal was inserted into the intergenic US1-US2 locus of BAC-R-LM113 (Fig. 2A, D). The recombinant Targeted Herpes Virus BAC, hereinafter referred to as THV_mICOSL, was transfected into SKOV3 cells and single plaques were isolated to harvest the viral particles (Fig. 2B). Viral particles were then purified by iodixanol gradient where single sharp band suggested the good productivity of infectious viral particles (Fig. 2C). Integrity of expression cassette and productivity of viral particles were respectively analyzed by Sanger sequence (not shown) and plaque assay (Fig. 2E). High titers and genome stability were assessed upon ten passages, demonstrating that mICOSL insertion did not affect virus yield (Fig. 2E).

**Fig. 3:**
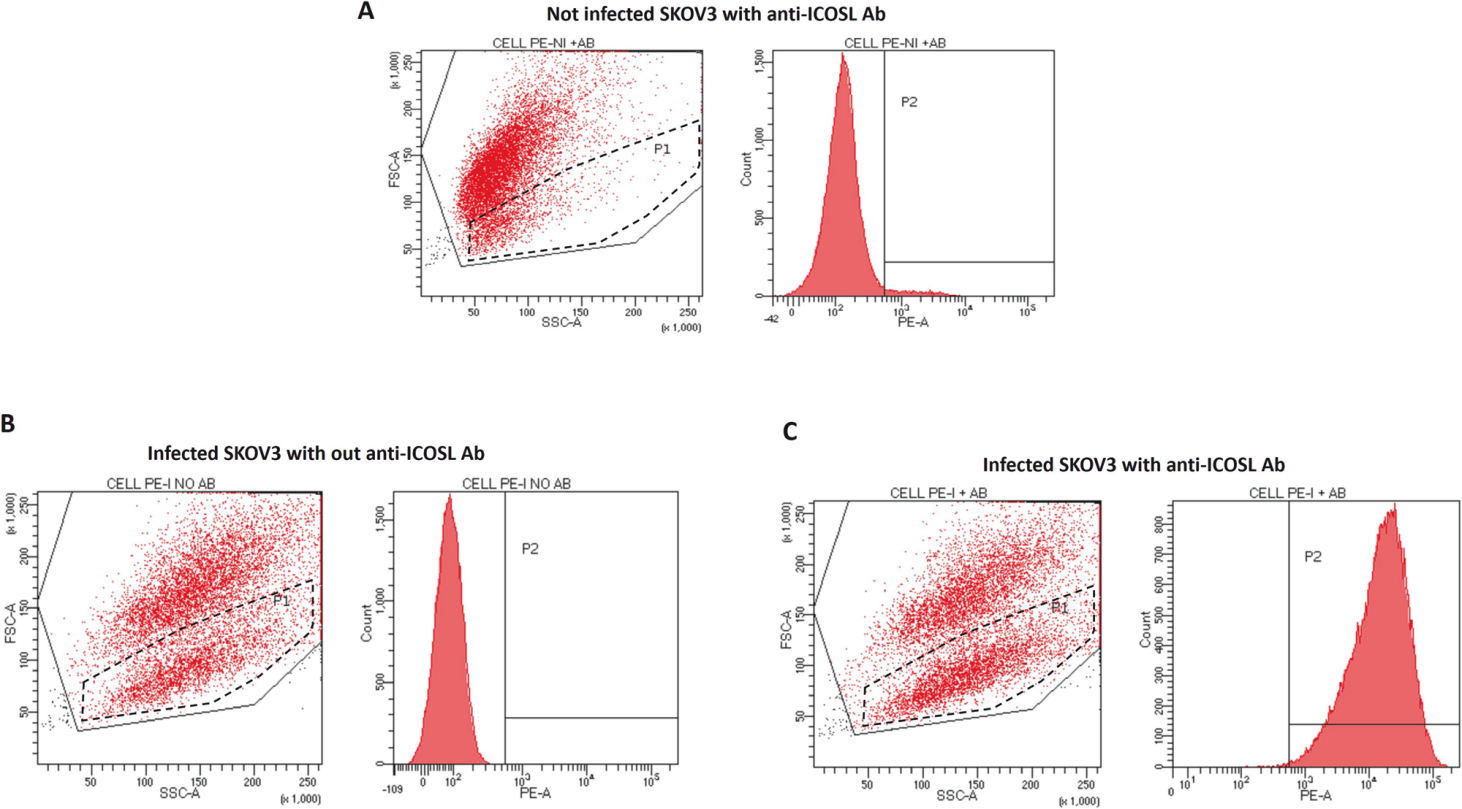
FACS assessment of mICOSL on THV_mICOSL infected SKOV3 cell line. **A** Not infected SKOV3 cells shows an absent ICOSL expression after staining with anti-mouse ICOSL. **B** Infected SKOV3 cells not stained with anti-ICOSL Ab in FSC-A value underlines a high percentage of infected, dead cells. **C** THV_ICOSL-infected SKOV3 cells were stained with anti-mouse ICOSL. Cells in P2 expressing mICOSL correspond to hundred percent of population. P1 population underlines a high percentage of infected, dead cells.

**Fig. 4:**
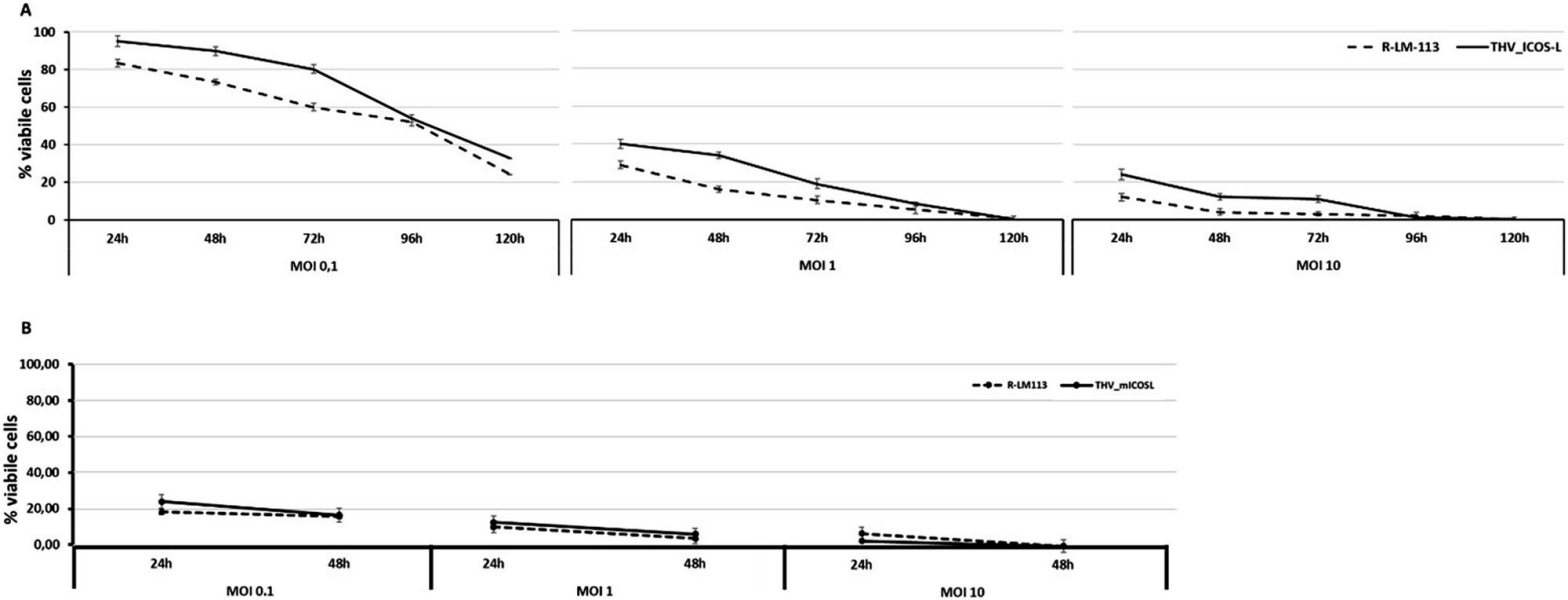
Comparison of cytotoxic effect of unarmed R-LM113 vs. THV_mICOSL on cancer cells. In (**A**) SKOV3 cells were respectively infected at 0.1, 1, 10 MOI and cell viability was assessed over 5 days post infection. In (**B**) the same experiment was performed in CT26 cells expressing hHER2. As expected, cytotoxicity was comparable between the unarmed and armed virus underlining the absence of a detrimental effect of arming. Percent of viable cells was calculated as percentage over not infected cells.

To assess the effectiveness of ICOSL display on cancer cell surface upon THV_mICOSL infection, flow cytometry analysis was performed on both non-infected and infected cells (Fig. 3). Murine ICOSL was expressed in near 100% cells of THV_mICOSL infected cells. The presence of small-size cell population in P1 was evident in FSC of infected cells, but not in non-infected cells (Fig. 3). This population was expected as a result of cell debris from infected cells. Control staining is reported in supplementary Figure 1. Infection was followed over 5 days in brightfield microscopy showing a dose-dependent cytotoxic effect (Fig. S2). A quantitative cytotoxicity assay was also performed by measuring the extracellular release of cytosolic Lactate dehydrogenase (LDH) as the measure of cell lysis. The cytotoxicity in human SKOV-3 tumor cells of THV_mICOSL was compared to the parental not armed R-LM113 demonstrating comparable levels of cell killing. This was true for each of the tested MOIs (0.1, 1, 10). In vitro cytotoxicity was also tested into murine cell line CT26 stably expressing human HER2. As expected, also in CT26 cell line, encoding ICOSL did not result in a hampered cytotoxicity compared to parental unarmed R-LM113 (Fig. 4B).

### In vivo efficacy of THV_mICOSL

**Fig. 5:**
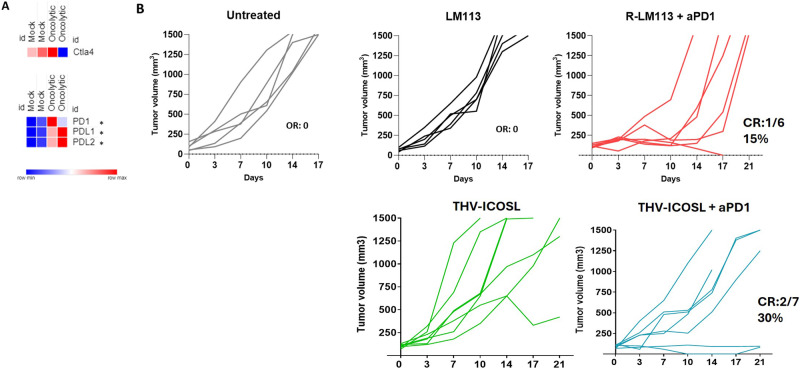
THV-ICOSL in combination with αPD1 slows LLC1-hHER2 tumor growth. Expression of CTLA4, PD1, PDL1, PDL2 was analyzed in the dataset described in Fig. 1 and reported as heatmap (**A**). (**B**) Efficacy data of depicted treatment in hHER2-transgenic mice challenged SC with LLC1-hHER2 cells. Intratumor treatment (IT) with PBS (Untreated), unarmed R-LM113 (5x 1E + 08), THV_mICOSL or R-LM113 (3x 5E + 07) alone or in combination with αPD1. Tumor volume was monitored over time. Lines in the graphs represent each individual tumor in a mouse. CR complete responder (also fibrotic residue).

**Fig. 6:**
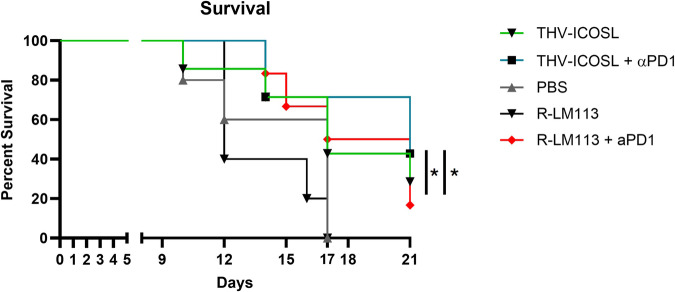
THV-ICOSL in combination with αPD1 improves mice survival. Survival curves of LLC1-hHER2 tumor bearing mice receiving the reported treatment. Mice were killed when tumor reached >1500 mm^3^. PBS n:5; R-LM113 n:5; R-LM113 *+* PD1 n:6; THV-ICOSL n:7; THV-ICOSL *+* PD1 n:7.

To further characterize tumor immunological reshape after oncolytic HSV, we also looked from the same dataset from pilot in vivo study described in Fig. 1, the expression of checkpoint inhibitors widely used into the clinic in combination with oncolytic viruses (i.e., CTLA4, PD1 and its ligands PDL1/PDL2) [36]. While CTLA4 expression was not affected by oncolytic viral infection, PD1 resulted highly upregulated in tumors after unarmed R-LM113 treatment (Fig. 5A). Interestingly, also its ligands PDL1 and PDL2 were significantly upregulated suggesting for the combination treatment of THV_mICOSL with a PD1 blockade (Fig. 5A). We and others have previous demonstrated the lack of efficacy of parental unarmed R-LM113 when tested in LLC1-hHER2 established (>100 mm^3^) tumor model [18, 25, 35]. The same Immunotherapy-refractory phenotype of LLC1 tumors was also validated with aPD1 monotherapy [25]. This lack of efficacy was due to a poor immune infiltration and immunosuppressive stimuli that make this tumor particularly aggressive and difficult to eradicate with traditional immunotherapeutic agents [37]. As expected, PBS (untreated) and R-LM113 treated mice showed a comparable tumor growth in line with our previous observations (Fig. 5B). R-LM113 was administered according as our gold standard schedule where 1E + 08 pfu were injected intratumorally on day 0, 2, 4, 7, 9 for a total of 5E + 08 pfu [25]. Here, to evaluate the impact of ICOSL expression, we de-escalated doses of R-LM113/aPD1 combination treatment and THV_mICOSL as monotherapy and in aPD1 combination arm. R-LM113 and THV_mICOSL were thus administered 3 times (day 0, 2, 5) with 5E + 07 pfu/injection for a total of 1.5E + 08 pfu. As expected R-LM113 monotherapy at 3x 5E + 07 did not exert any tumor control (data not shown). THV_mICOSL monotherapy allowed to delay tumor growth in three out of seven mice (Fig. 5B) with a trend of tumor delay like the combination group R-LM113/aPD1. When combined with anti PD-1 mAb, THV_mICOSL further prolonged control of tumor size and induced near complete response in 2 out of 7 animals (Fig. 5B). Survival analysis confirmed the benefit of ICOSL arming in THV_mICOSL over unarmed R-LM113. Also, aPD-1 combination treatment showed the significant improvement in survival (Fig. 6).

### Human THV_ICOSL activates human T lymphocytes

**Fig. 7:**
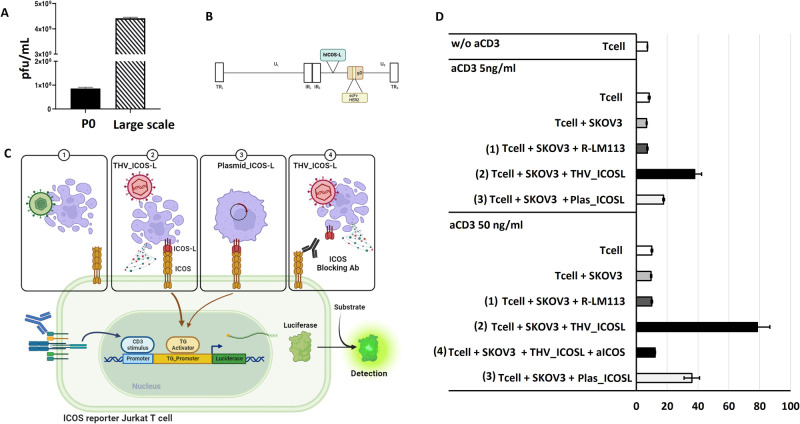
In vitro validation of biological activity of human ICOSL armed THV. **A** Titers of transfection (P0) and purified THV_hICOSL virus on iodixanol gradient. **B** The human ICOSL coding sequence is inserted into intergenic region US1-US2 in the background of unarmed THV. The scFv targeting hHER2 is shown in glycoprotein D. **C** Schematic representation of ICOS Blockade Bioassay used to measure the potency and stability of ligands that binds ICOS receptor. Jurkat T cells, endogenously express TCR/CD3 and are engineered to express human ICOS and a NanoLuc luciferase reporter driven by ICOS and TCR/CD3 pathway-dependent response elements. **D** Activation of Jurkat T cells in coculture with SKOV3 tumor cells infected with THV empty (R-LM113) and THV_hICOSL at MOI 0,1 pfu/cell.

Promising results obtained with murine version of THV virus encoding mICOSL spurred us to generate a human analog expressing human ICOSL hereinafter referred to as THV_hICOSL. THV_hICOSL BAC DNA was rescued in SKOV3 cells to obtain viral seed (P0). Large scale infection and purification was performed for further in vitro validation (Fig. 7A). Except for the transgene (human in the place of murine ICOSL), the layout of THV_mICOSL was maintained into THV_hICOSL (Fig. 7B). To assess the potential clinical activity of a THV expressing the human orthologue of mICOSL, we constructed an in vitro co-culture assay to reproduce the immunological synapse between THV-infected cancer cells and human T lymphocytes (Fig. 7C). We implemented the Jurkat T cell line engineered to express nanoLuc under the control of transcriptional elements responsive to TCR/CD3 plus ICOS stimulation (Fig. 7C). Anti CD3 mAb was used at two different concentrations (5 and 50 ng/ml) to trigger TCR/CD3 first signal in Jurkat T cells. As negative controls, Jurkat cells were cultured alone, in the presence of aCD3 mAb (5 and 50 ng/ml) or cocultured with uninfected tumor cells. As expected, T lymphocytes w/o CD3, or even in the presence of anti CD3 mAb did not produce reporter gene signal. Co-culturing of Jurkat with THV_hICOSL-infected tumor cells revealed the ability of THV_hICOSL to strongly activate T cells. This effect was validated with both 5 and 50 ng/ml aCD3. On the contrary, no activation was detected in coculture of Jurkat with unarmed R-LM113-infected cells. The co-culture of Jurkat T cells with tumor cells transiently transfected with a hICOSL expressing plasmid were used as a positive control. Interestingly, expression of hICOSL by DNA plasmid exerted a significantly lower activation of T lymphocytes compared to hICOSL delivered as payload into the OV. This was presumably due to immunogenic cell death and the release of DAMPs and PAMPs from infected cells [11]. The specificity and ICOSL dependency were further validated by using an ICOS-blocking antibody in the coculture of THV_hICOSL-infected cells and Jurkat T cells. The presence of saturating concentration of a blocking anti-hICOS mAb abrogated T cell activation, demonstrating the direct link between display of THV-encoded ICOSL of cancer cell surface and T cell activation (Fig. 7D).

## Discussion

The concept of tumor therapy with viruses has been strengthened by hundreds clinical trials performed since as far back as 1949. Despite enthusiasm and intriguing mechanism of action of these unconventional cancer treatments, after more than 70 years only T-VEC has been approved by FDA. After years of thinking at OVs as a “magic bullet” infecting, replicating, and destroying cancer cells by direct cell lysis, it is nowadays accepted by scientific community that a small percentage of tumor cells are actually cleared out from the tumor mass, and that most of the therapeutic activity relies on in situ vaccination effect. Thanks to the ability to recruit and activate anti-tumor-specific immune cells, OVs entered the class of cancer immunotherapeutics. Elicitation of antitumor immune response allows to disregard the limitation of intratumor delivery since distal tumor sites can be cured by abscopal effect. While such oncolytic platforms are more prone to be administered systemically, elicitation of abscopal antitumor effect must be obtained with oncolytic viruses that can arise issues if delivered systemically. This is the case of Herpes simplex virus where pre-existing immunity and/or neurotropism limit systemic delivery [38]. While the preclinical efficacy of OVs has been proved with many different viral platforms (DNA, RNA viruses) and models, their clinical transition moves slowly and patchily. Many causes contribute to this fluctuating interest in oncovirotherapy made of successes and unpredictable failures [39]. This course can result from (i) the highly suppressive tumor indication often targeted by OVs (e.g., glioblastoma, advanced stage indications); (ii) safety issues that spurred scientists to use weakened viruses (highly attenuated); (iii) the line of treatment, in which clinical trial participants have typically undergone several rounds of potentially immunosuppressive therapies (e.g., chemotherapy) before being treated with oncovirotherapy [40]. Despite suboptimal clinical success, those experimentations have convinced regulatory agencies that OVs are safe and can be improved by different strategies. One strategy is preserving virulence, without affecting the safety profile. Systems biology allowed to define tumor restricted promoters, tumor-associated antigens, tumor-deficient miRNAs and many different other cancer-altered regulation mechanisms, which can be exploited to restrict OVs’ tropism while retaining their virulence (e.g., retargeting to TAA, restricting viral gene transcription or translation by tumor selective promoters of miRNAs) [16–19]. These therapeutical designs are increasing the use of OVs especially in those tumors defined as immune hostile (e.g., recurrent GBM), recently object of successful clinical outcome with CAN-3110 oncolytic HSV-1 (NCT03152318) [41]. The second strategy to enhance OV efficacy is arming them with immunomodulatory genes, encoded into the OV genome, in order to exert an intratumor gene therapy. Long term results from OPTiM phase III trial with T-VEC, the first armed OV approved by FDA and EMA in melanoma, have revealed the advantage of HSV-1 expressing GM-CSF *vs* GM-CSF administered in its proteinaceous form. Advantages of vectorizing immomodulatory agents as “cis” agent into OVs has been recently reviewed by Leonard W Seymour, who underlines how localized expression mitigates the systemic toxicity while enhance penetration in the tumor core [42].

Beside GM-CSF, many cytokines, chemokines and co-stimulatory molecules have been vectorized as payloads into OVs, often in the absence of any supportive rationale [21]. Many costimulatory agents have been adopted as payload into different DNA or RNA-based OVs (41BB-L, OX40L, CD40L, CD80, GITRL, LIGHT, CD70) [21, 43–45]. Nowadays, the tumor complexity can be resolved up to single cell level, offering the opportunity of data-driven arming and combination strategies. Here, we aimed to combine a non-attenuated, tumor-restricted HER2-targeted herpes virus with a novel data-driven arming approach to identify an appropriate costimulatory agonist to encode as a payload. By transcriptomic characterization of tumors, we demonstrate a significant upregulation of ICOS post oncolytic herpes virus treatment. On the contrary, its endogenous ligand (ICOSL) resulted downregulated. This observation spurred us to encode ICOSL into the fully virulent, HER2-targeted herpes virus to trans-complement the ligand-receptor pair. ICOSL-armed targeted herpes virus (THV) improved in vivo efficacy and synergized with PD-1 blockade. Co-culture assay with human T cells demonstrate the ability of THV-ICOSL-infected cells to activate T lymphocytes. While we reported the CD8 dependency of R-LM113 immunotherapeutic efficacy, further future analysis will be dedicated the dissect the mechanism of action of encoded ICOSL. By searching into several available datasets and published results, we identified ICOS as the most recurrent upregulated costimulatory receptor after oncolytic virus treatment. This result is consistent in preclinical and clinical settings and with very different oncolytic viral platforms including but not limited to clinical stage vaccina virus JX-594 (Pexa-vec), Reovirus (Reolysin, Pelareorep), multi-cytokine armed vaccinia virus hIL-7/mIL-12-VV, new castle disease virus (NDV), HSV-1 canerpaturev (C-REV, formerly HF10) where high levels of ICOS from tumor biopsies allowed the stratification of responders *vs* non-responders patients [29, 46–48]. Despite that, ICOS has never been implemented in clinical oncolytic viruses. Our data, together with these aforementioned clinical and preclinical data give a piece of advice for rapid clinical translation of ICOSL as vectorized costimulatory gene into many different oncolytic virus platforms.

## Matherials and methods

### Cell cultures, manipulation, and characterization

SKOV3 and Jurkat T cells were cultured with RPMI 1640 Medium GlutaMAX^TM^ Supplement (GibcoTM, Thermo Fisher Scientific, Waltham, MA, USA) in a humidified atmosphere containing 5% CO2 at 37 °C. The media was supplemented with 10% heat-inactivated fetal bovine serum and 50 UI/mL penicillin, 50 μg/mL streptomycin (GibcoTM, Thermo Fisher Scientific, Waltham, MA, USA). Cell lines were purchased from the American Type Culture Collection (ATCC) and from Promega (Madison, WI, USA). Cytotoxicity was assessed at indicated timepoint and percentage of live cells was normalized to not infected cells. All infections were reported as average of three biological replicate.

### Immune profiling panel by Nanostring and real-time PCR

Female heterozygous C57 B6.Cg-Pds5b<Tg(Wap-ERBB2)229Wzw>/J mice were used for in vivo study according to the Ethical Guidelines and as approved by Italian ministry of health. 5 × 10^5^ LLC-ERBB2 LLC1 cells stably expressing human HER2 were subcutaneously implanted on the right flank. Ten days after implantation 1x10E8 infectious viral particles of unarmed R-LM113 were administered in randomized tumors >100 mm^3^. Same volume (50 μl) of PBS was used in control untreated mice. The day after treatment, mice were killed, tumors excised and stored in RNA later. Bulk RNA was extracted and analyzed by PanCancer immune profiling panel by Nanostring NCounter. Also, RNA was reverse-transcribed by using ImProm-II Reverse Transcriptase (Promega, Madison, WI, USA). Differentially expressed gene of interest were validated by Real Time PCR using SYBR Green PCR Mastermix (Applied Biosystem, Foster City, CA, USA) with ICOS and ICOSL specific probes. The relative abundance of target RNAs was evaluated in relation to actin transcript by ΔΔCt method [49].

### Retargeted virus generation and production

The sacB/ampR/lacZ recombineering system was exploited to modify HSV-1 vectors as previously reported [18]. Briefly, THV viruses were generated by recombineering starting from a wild type strain F virus inserted into a BAC plasmid in SW102 E.Coli. Retargeted HSV-1_BAC carrying CMV promoter and polyA into US1–US2 intergenic locus was generated as reported in the work of Gentile et al. [10]. The cassette encoding Amp/SacB/LacZ, with homology arms to the region to be engineered, was inserted between CMV and polyA in the first step of recombineering by electroporation of SW102 cells containing the BAC-HSV-1. Bacteria cells were plated on LB agar plus 12.5 μg/mL chloramphenicol, 20 μg/mL ampicillin, 80 μg/mL X-gal, and 200 uM IPTG. Positive clones (Blue, Amp resistance with functional SacB gene) were used for the second step of recombineering to replace the selection cassette with the transgene of interest. The negative selection was performed on plates containing sucrose. To verify sequences’ integrity of recombinant THV_mICOSL and THV_hICOSL, the expression cassette encoding mICOSL and hICOSL was amplified by PCR performed on DNA extracted from rescued viral particles at P0. The amplicons were sequenced by Sanger sequencing. The following oligonucleotides were used for recombineering:

F_StepI: ctggctagcgtttaaacgggccctctagactcgagcggccgcacgccaccacccctatttgtttatttttct

R_StepI: ggcaactagaaggcacagtcgaggctgatcagcggtttaaacttaagcttttatttgttaactgttaattgtc

F_StepII: ctggctagcgtttaaacgggccctctagactcgagcggccgcacgccaccatgcagctgaagtgcccctg

R_StepII: ggcaactagaaggcacagtcgaggctgatcagcggtttaaacttaagctttcattaggcgtgatctgtca

### Viral rescue, production and titration

For viral rescue SKOV3 cells were transfected with BAC HSVs DNA by Lipofectamine 2000 (Life Technologies, Carlsbad, CA, USA) and grown until full cytopathic effect (CPE) was reached. Viral particles were amplified by serial passages, then purified from conditioned supernatant by iodixanol gradient and were titrated by plaque assays by 10-fold scaling dilutions. After each passage, infected SKOV-3 cells were sonicated to extract viral particles and its titration was made by plaque assays by 10-fold scaling dilutions.

### FACS analysis

FACS analysis was performed to assess ICOSL expression on cell surface, after THV_mICOSL infection, according to the procedure previously described [50]. Briefly, SKOV-3 cells were detached by using PBS EDTA 5 mM and washed 2 times with PBS. Cells were stained with anti-ICOSL antibody and analyzed by cell-sorter Becton Dickinson FACSAria. THV_mICOSL cytotoxicity was assessed through live cell percentage up to 5 days post infection at three different MOI (0.1, 1, 10) by counting live cells on the non-infected count.

### In vivo experiments

Authorization/ethics approval for in vivo studies was approved by Italian ministry of health n° 726/2022-PR (A69A0.107). Female heterozygous C57 B6.Cg-Pds5b<Tg(Wap-ERBB2)229Wzw>/J mice were used for in vivo study. 5 × 10^5^ LLC-ERBB2 cells were implanted in the right flank of mice. Ten days after implantation tumors became established (>100 mm^3^). Mice were randomized according to tumor size and treated with unarmed R-LM113 or with THV_mICOSL virus. R-LM113 was administered five times, on day 0, 2, 4, 7, 9 at concentration of 1x10E8 infectious viral particles for each intratumor injection for a total of 5X10E8 pfu. THV_mICOSL was administered three times (day 0, 2, 4) in a dose de-escalation compared to unarmed R-LM113. 5 × 10^7^ pfu were administered in each injection for a total of 1.5X10E8 pfu. THV_mICOSL was also tested in combination with systemic (intraperitoneal) 200 μg α-mPD-1 (BioXcell, clone RMP114) on day 0, 3, 7, 10. Tumor growth and mice survival were assessed up to 17 days. For ethical concerns, animal death was not the endpoint of experimentation; for this reason, when tumors reached 1500 mm^3^, mice were killed. Mouse killing was included in Kaplan–Meier survival curve.

### ICOSL blockade assay

In vitro hICOSL activity was assessed through the ICOS blockade bioassay Promega (Promega, Madison, WI, USA). At day one SKOV-3 cells were plated in black mw96 10 000cell/well. The next day we infected the cells with THV-hICOSL or R-LM-113 or transfected them with plasmid_ICOSL. At day 3 we added, in co-culture with SKOV-3, Jurkat T cells expressing ICOS, an endogenous TCR/CD3 and a NanoLuc (NL) luciferase reporter driven by ICOS and TCR/CD3 pathway-dependent response elements. At the same time, we also added an mAb Anti CD3 Ultra-LEAF™ Purified anti-human CD3 Antibody (BioLegend, San Diego, CA, USA) at two different concentrations (5 ng/ml and 50 ng/ml) in order to trigger CD3 activation in Jurkat T cells. After 6 h of incubation, we performed Luciferase Assay according to Bio-Glo-NL™ Luciferase Assay.

## Supplementary information

Supplementary information

## Data Availability

All data, cell line models, constructs, and viral vectors are available upon request to Emanuele Sasso emanuele.sasso@unina.it
