# Supplementary material for: Integrating system biology and intratumor gene therapy by trans-complementing the appropriate co-stimulatory molecule as payload in oncolytic herpes virus
Source: Cancer Gene Ther. 2024 Jun 5;31(9):1335–43. doi: 10.1038/s41417-024-00790-8 (PMC11405262; doi:10.1038/s41417-024-00790-8)
Supplement: Supplementary file 1 — Supplementary information [file 41417_2024_790_MOESM1_ESM.docx]

**Integrating System Biology and Intratumor Gene Therapy by Trans-Complementing the Appropriate Co-Stimulatory Molecule as Payload in Oncolytic Herpes Virus.**

**A. Finizio^1,2,*^, P. Pagano^1,2,*^, A. Napolano^1,2^, G. Froechlich^1,2^, L. Infante^3^, A. De Chiara^1,2^, S. Amiranda^1^, E. Vitiello^1^, S. Totaro^1,2^, C. Capasso^1,2^, M. Raia^2^, A.M. D’Alise^3^, P. de Candia^1^, N. Zambrano^1,2^ and E. Sasso^1,2,4^**

^1^ Dipartimento di Medicina Molecolare e Biotecnologie Mediche, Università degli Studi di Napoli Federico II, Via Pansini 5, 80131 Napoli, NA, Italy. ^2^ CEINGE Biotecnologie Avanzate Franco Salvatore S.C.aR.L., Via Gaetano Salvatore 486, 80145 Naples, Italy. ^3^ Nouscom S.R.L., Rome, Italy. ^4^ ImGen-T Srl, Viale del Parco Carelli, Napoli, NA, Italy. * These authors contributed equally.

Correspondence to Emanuele Sasso emanuele.sasso@unina.it


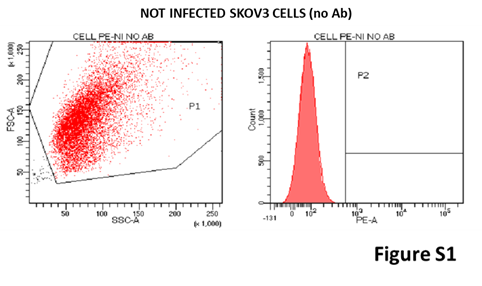


**Supplementary figure 1.** FACS analysis of control uninfected cells w/o staining.


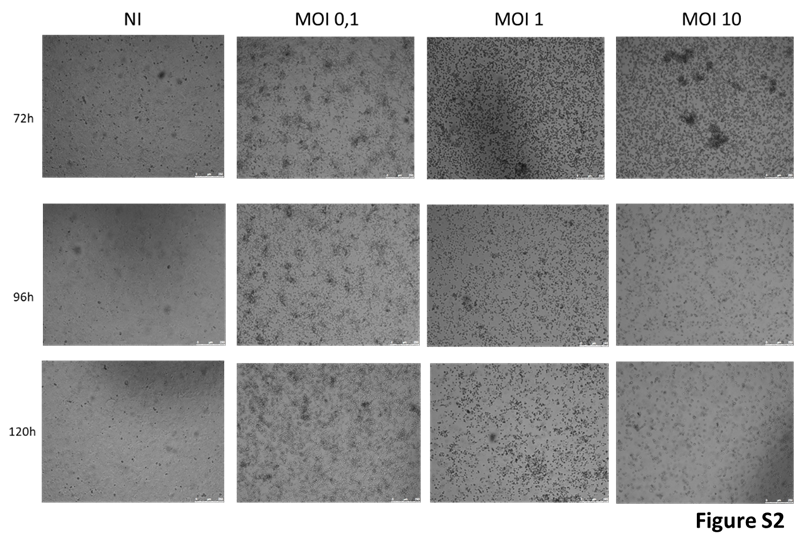


**Supplementary Figure 2.** Bright Field microscopy of THV_ICOSL-infected cells in comparison with uninfected (not infected NI) cells. The three different MOI of infection and timecourse analysis are depicted in figure.
